# Contribution of Common Genetic Variants to Obesity and Obesity-Related Traits in Mexican Children and Adults

**DOI:** 10.1371/journal.pone.0070640

**Published:** 2013-08-08

**Authors:** Paola León-Mimila, Hugo Villamil-Ramírez, Marisela Villalobos-Comparán, Teresa Villarreal-Molina, Sandra Romero-Hidalgo, Blanca López-Contreras, Roxana Gutiérrez-Vidal, Joel Vega-Badillo, Leonor Jacobo-Albavera, Carlos Posadas-Romeros, Adrián Canizalez-Román, Blanca Del Río-Navarro, Francisco Campos-Pérez, Victor Acuña-Alonzo, Carlos Aguilar-Salinas, Samuel Canizales-Quinteros

**Affiliations:** 1 Unidad de Genómica de Poblaciones Aplicada a la Salud, Facultad de Química, Universidad Nacional Autónoma de México (UNAM)-Instituto Nacional de Medicina Genómica (INMEGEN), Mexico City, Mexico; 2 Unidad de Biología Molecular y Medicina Genómica, Instituto Nacional de Ciencias Médicas y Nutrición “Salvador Zubirán” (INCMNSZ), Mexico City, Mexico; 3 Departamento de Genómica Computacional, INMEGEN, Mexico City, Mexico; 4 Laboratorio de Enfermedades Cardiovasculares, INMEGEN, Mexico City, Mexico; 5 Departmento de Endocrinología, Instituto Nacional de Cardiología Ignacio Chávez (INCICh), Mexico City, Mexico; 6 Facultad de Medicina, Universidad Autónoma de Sinaloa, Culiacán, Mexico; 7 Departamento de Alergia e Inmunología Clínica, Hospital Infantil de México Federico Gómez, Mexico City, Mexico; 8 Clínica de Obesidad, Hospital Rubén Leñero, Mexico City, Mexico; 9 Escuela Nacional de Antropología e Historia, Mexico City, Mexico; 10 Departmento de Endocrinología y Metabolismo, INCMNSZ, Mexico City, Mexico; Children's National Medical Center, Washington, United States of America

## Abstract

**Background:**

Several studies have identified multiple obesity-associated loci mainly in European populations. However, their contribution to obesity in other ethnicities such as Mexicans is largely unknown. The aim of this study was to examine 26 obesity-associated single-nucleotide polymorphisms (SNP) in a sample of Mexican mestizos.

**Methods:**

9 SNPs in biological candidate genes showing replications (*PPARG*, *ADRB3*, *ADRB2*, *LEPR*, *GNB3*, *UCP3*, *ADIPOQ*, *UCP2,* and *NR3C1*), and 17 SNPs in or near genes associated with obesity in first, second and third wave GWAS (*INSIG2*, *FTO*, *MC4R*, *TMEM18*, *FAIM2*/*BCDIN3*, *BDNF*, *SH2B1*, *GNPDA2*, *NEGR1*, *KCTD15*, *SEC16B/RASAL2*, *NPC1*, *SFRF10/ETV5*, *MAF*, *PRL*, *MTCH2*, and *PTER*) were genotyped in 1,156 unrelated Mexican-Mestizos including 683 cases (441 obese class I/II and 242 obese class III) and 473 normal-weight controls. In a second stage we selected 12 of the SNPs showing nominal associations with obesity, to seek associations with quantitative obesity-related traits in 3 cohorts including 1,218 Mexican Mestizo children, 945 Mexican Mestizo adults, and 543 Indigenous Mexican adults.

**Results:**

After adjusting for age, sex and admixture, significant associations with obesity were found for 6 genes in the case-control study (*ADIPOQ*, *FTO*, *TMEM18*, *INSIG2*, *FAIM2*/*BCDIN3* and *BDNF*). In addition, *SH2B1* was associated only with class I/II obesity and *MC4R* only with class III obesity. SNPs located at or near *FAIM2*/*BCDIN3*, *TMEM18*, *INSIG2*, *GNPDA2* and *SEC16B*/*RASAL2* were significantly associated with BMI and/or WC in the combined analysis of Mexican-mestizo children and adults, and *FTO* locus was significantly associated with increased BMI in Indigenous Mexican populations.

**Conclusions:**

Our findings replicate the association of 8 obesity-related SNPs with obesity risk in Mexican adults, and confirm the role of some of these SNPs in BMI in Mexican adults and children.

## Introduction

The prevalence of obesity is rapidly increasing worldwide [1]. According to the 2012 National Health and Nutrition Survey in the Mexican population, the prevalence of overweight and obesity is 71.2% in adults aged above 20 years and 34.4% in children [2]. Moreover, according to the 2002 Mexican Family Life survey, 59% of indigenous Mexican individuals are overweight or obese [3]. Hereditability of obesity has been estimated as high as 70% [4]. Using a biological candidate gene approach, more than 127 genes have been associated with obesity and/or obesity-related phenotypes [5]; while genome-wide association studies (GWAS) have identified more than 120 genes (the vast majority not previously identified as biological candidates) associated with obesity mainly in European populations [6].

Although associations of many common genetic variants with obesity have been replicated mainly in several European and Asian populations [5], [6] there are still few studies in the Mexican population [7]–[10]. This population resulted from recent admixture mainly of indigenous Mexican and European populations [11], and thus genetic variants that are common in Europeans are likely to be part of the genetic architecture of obesity in Mexicans. Using a case control design, we sought to assess the contribution of two different sets of SNPs with obesity in Mexican Mestizo adults: 9 SNPs in biological candidate genes showing replications in at least 10 studies (*PPARG*, *ADRB3*, *ADRB2*, *LEPR*, *GNB3*, *UCP3*, *ADIPOQ*, *UCP2*, and *NR3C1*) [5] and 17 SNPs in or near genes associated with obesity in first, second and third wave GWAS (*INSIG2*, *FTO*, *MC4R*, *TMEM18*, *FAIM2*/*BCDIN3*, *BDNF*, *SH2B1*, *GNPDA2*, *NEGR1*, *KCTD15*, *SEC16B*/*RASAL2*, *NPC1*, *SFRF10*/*ETV5*, *MAF*, *PRL*, *MTCH2*, and *PTER*) [12]–[18]. In a second stage we selected of the SNPs showing nominal associations with obesity, to seek associations with quantitative obesity-related traits in 3 distinct cohorts of Mexican Mestizo children and adults, as well as Indigenous Mexicans.

## Research Design and Methods

### Case-control Study

The case-control study included 1 156 unrelated Mexican-Mestizos. Control, class I and class II obesity individuals were workers from several Governmental Institutions in Mexico City, including Instituto Nacional de Neurología y Neurociencias Manuel Velasco Suárez, Centro Médico Nacional Siglo XXI, Instituto Nacional de Ciencias Médicas y Nutrición Salvador Zubirán (INCMNSZ), Universidad Nacional Autónoma de México, and Universidad Autónoma Metropolitana. Individuals with class III obesity were outpatients from Obesity Clinics at the INCMNSZ and the Dr. Rubén Leñero General Hospital. All participants were aged 18 to 82 years, without chronic disease that may compromise body weight (including cancer, HIV infection and thyroid disorders); 473 were non-diabetic normal weight subjects (BMI >18.5 and ≤25 kg/m^2^); 441 were obese class I/II individuals (BMI ≥30 kg/m^2^ and <40 kg/m^2^), and 242 were obese class III individuals (IMC ≥40 kg/m^2^) described by Villalobos-Comparán et al. [9]. Anthropometric characteristics of the subjects are summarized in Table S1. All participants provided written informed consent prior to the inclusion in the study.

### Cohort Studies

This study analyzed three independent cohorts. The first group included 945 unrelated Mexican-mestizos aged 18–82 years, recruited at their work-sites at different governmental institutions in Mexico City (including the Instituto Nacional de Neurología y Neurociencias Manuel Velasco Suárez, Centro Médico Nacional Siglo XXI, Instituto Nacional de Ciencias Médicas y Nutrición Salvador Zubirán, Universidad Nacional Autónoma de México, and Universidad Autónoma Metropolitana-Iztapalapa), 788 described by Villalobos-Comparán et al. [8]. A total of 441 of these subjects were also included in the case-control study described above.

The second group included 1 218 healthy unrelated school-aged Mexican-Mestizo children (595 boys and 658 girls) aged 6–15 years, recruited from a summer camp for children of employees of the Mexican Health Ministry (Convivencia Infantil 2008, Secretaria de Salud) and from a public junior high school in Mexico City, previously described by Flores-Dorantes et al [19]. A parent or guardian of each child signed the consent form for participation.

The third group included 543 unrelated individuals aged over 18 years, belonging to 4 indigenous groups from rural communities: 77 Seris from Sonora located in Northern Mexico, 271 Nahuas and 112 Totonacas from Puebla in East-central Mexico, and 83 Zapotecs from Oaxaca in Southeastern Mexico. All individuals in this group, their parents and grandparents recognized themselves as indigenous, had been born and lived in their home communities, and spoke their native language. Blood samples were drawn with the permission of local authorities and a translator was used as needed.

This study was conducted according to the principles expressed in the Declaration of Helsinki and was approved by the Ethics Committees of participant institutions.

### Anthropometric and Biochemical parameters

Anthropometric measurements were determined following the procedures recommended by Lohman et al. [20] and included weight, height, waist circumference (WC) and hip circumference. All instruments were calibrated following the standard methods of the manufacturers. BMI was calculated as weight in kilograms divided by the square of height in meters. In adults, obesity status was determined according to WHO (World Health Organization) criteria [21]. In children, BMI z-scores were calculated using age and sex specific BMI reference data, as recommended by the Centers for Disease Control and Prevention [22]. Biochemical parameters including fasting glucose, insulin, total cholesterol, HDL-C and triglycerides serum levels were measured as previously described [23]. Homeostasis model assessment of B-cell function (HOMA-B) was estimated using a computer model [24].

### SNP selection and genotyping

Genomic DNA was isolated from peripheral blood white cells using a commercial kit based on the salt fractionation method (QIAmp 96 DNA Blood Kit, Quiagen, Hilden, Germany). A total of 26 SNPs in or near genes previously associated with obesity risk in other populations were genotyped in cases and controls: 12 SNPs were selected from 9 biological candidate genes including rs3856806 (*PPARG*), rs4994 (*ADRB3*), rs1042719 (*ADRB2*), rs1137101 (*LEPR*), rs5443 (*GNB3*), rs1800849 (*UCP3*), rs2241766 (*ADIPOQ*), rs659366 (*UCP2*) and rs56149945 (*NR3C1*); while the remainder 17 SNPs were selected from first, second and third wave GWAS reports and included rs7566605 (*INSIG2*), rs9939609 (*FTO*), rs17782313 (*MC4R*), rs6548238 (*TMEM18*), rs7138803 (FAIM2/BCDIN3), rs6265 (*BDNF*), rs7498665 (*SH2B1*), rs10938397 (*GNPDA2*), rs2815752 (*NEGR1*), rs29941 (*KCTD15*), rs10913469 (*SEC16B*/*RASAL2*), rs1805081 (*NPC1*), rs7647305 (SFRS10/ETV5), rs1424233 (MAF), rs4712652 (PRL), rs10838738 (*MTCH2*) and rs10508503 (*PTER*).

Because the Mexican-Mestizo population is admixed, ancestry informative markers (AIMs) were used to assess whether any association could be confounded by population stratification. A panel of 10 AIMs (rs4884, rs2695, rs17203, rs2862, rs3340, rs722098, rs203096, rs223830, rs1800498, and rs281478) distinguishing mainly Amerindian and European ancestry (δ>0.44) [8] were genotyped in the case-control sample.

Genotyping was performed using TaqMan Probes (ABI Prism 7900HT Sequence Detection System; Applied Biosystems) and/or KASPAR assays (Kbioscience, U.K. http://www.kbioscience.co.uk/). Call rate exceeded 95% for all SNPs tested, with no discordant genotypes in 10% of duplicate samples. In addition, all samples were genotyped for rs756605 (*INSIG2*) using both methods, finding no discordant genotypes. Deviation from Hardy–Weinberg equilibrium was not observed for any SNPs in any group (*P*>0.05).

### Statistical analyses

Associations of each SNP with obesity were tested using logistic regression analysis. The AdmixMap program was used to test the possible effect of population stratification on associations with obesity [25], [26]. Because the Mexican-Mestizo population derived mainly from Amerindian and European (Spanish) populations, the model included two primary parental populations. Admixmap fits a logistic regression model of the trait on individual admixture, and it allows the inclusion of covariates such age and sex. All associations were tested for additive, dominant and recessive inheritance models, reporting the most significant. To assess the combined effect of risk alleles, we calculated genotype score counting the number of risk alleles through a logistic model adjusted for age, sex and admixture.

All obesity-related quantitative variables were transformed to normal distribution with a mean of zero and standard deviation (SD) of one in each study separately using inverse normal transformation. Effect sizes were compared across traits and age groups using linear regression analysis. Combined association tests for obesity quantitative traits in children and adult populations and among Indigenous populations were conducted using a Mantel–Haenszel-like model. The combined estimated effect was computed as weighted average of the individual estimated effects using weights proportional to the inverse of the standard errors squared [27]. Cochran’s Q test was used to analyze heterogeneity among study populations [28]. All statistical analyses were performed using SPSS (version 16.0; Chicago, IL). Because the majority of the SNPs analyzed were well validated variants, a *P*-value threshold <0.05 was used for declaring significant association.

## Results

### Case-control study

Of the 9 biological candidate variants, only *NR3C1* rs56149945 showed a very low minor allele frequency (<0.01), and was thus excluded from the analyses. Only 2 of the 8 SNPs previously associated with obesity using this approach showed significant association in the Mexican population. *ADIPOQ* rs2241766 showed a nominal significant association with overall obesity (OR 2.34, *P_rec_*  = 0.033, recessive model), and *UCP3* rs1800849 was significantly associated only with class I/II obesity (OR 1.33, *P_add_*  = 0.050, additive model; Table 1).

**Table 1:** Associations of Candidate SNP loci with Obesity in the Mexican Population.

					Overall obese vs. normal weight			Class I/II obese vs. normal weight			Class III obese vs. normal weight			
Nearest gene	Chr	SNP	Ref allele	Test allele	OR (95 %CI)	*^a^P*	*^b^P*	OR (95 % CI)	*^a^P*	*^b^P*	OR (95% CI)	a*P*	b*P*	*P*-Het
**Biological candidates**														
*ADIPOQ*	3	rs2241766	G	G	**2.34 (1.07–** **5.12**)	**0.033**	**0.033**	**2.76 (1.23–** **6.19)**	**0.014**	**0.017**	1.26 (0.37– 4.22)	0.713	0.722	s0.289
*UCP3*	11	rs1800849	C	C	1.25 (0.97– 1.62)	0.085	0.086	**1.33 (1.00–** **1.77)**	**0.050**	0.061	1.09 (0.77– 1.56)	0.632	0.667	0.398
*ADRB3*	12	rs4994	T	C	1.15 (0.89– 1.48)	0.297	0.316	1.16 (0.88– 1.52)	0.293	0.412	1.13 (0.81– 1.58)	0.480	0.341	0.910
*PPARG*	3	rs3856806	C	T	1.18 (0.86– 1.62)	0.313	0.315	1.14 (0.81– 1.61)	0.442	0.447	1.28 (0.85– 1.94)	0.240	0.268	0.678
*ADRB2*	5	rs1042719	G	C	1.09 (0.89– 1.34)	0.408	0.447	1.12 (0.90– 1.39)	0.326	0.421	1.03 (0.78– 1.36)	0.853	0.761	0.638
*UCP2*	11	rs659366	C	T	1.08 (0.89– 1.33)	0.436	0.438	1.06 (0.86– 1.32)	0.581	0.581	1.14 (0.87– 1.49)	0.357	0.365	0.709
*LEPR*	1	rs1137101	G	A	1.05 (0.85– 1.30)	0.625	0.625	1.05 (0.83– 1.31)	0.700	0.729	1.06 (0.80– 1.41)	0.668	0.694	0.927
*GNB3*	12	rs5443	T	C	1.02 (0.84– 1.23)	0.853	0.857	0.93 (0.76– 1.15)	0.517	0.513	1.21 (0.92– 1.59)	0.176	0.139	0.142
**GWAS candidates**														
*FTO*	16	rs9939609	A	A	**1.42 (1.15–** **1.76)**	**0.001**	**0.003**	1.19 (0.93– 1.52)	0.174	0.146	**1.88 (1.44–** **2.45)**	**3.4×10^−6^**	**1.8×10^−5^**	**0.031**
*TMEM18*	2	rs6548238	C	C	**1.57 (1.17–** **2.12)**	**0.003**	**0.001**	**1.74 (1.23–** **2.46)**	**0.002**	**0.004**	**1.29 (0.87–** **1.91)**	**0.200**	**0.028**	0.260
*INSIG2*	2	rs7566605	C	G	**1.33 (1.08–** **1.63)**	**0.006**	**0.006**	**1.38 (1.10–** **1.74)**	**0.005**	**0.006**	1.23 (0.92– 1.63)	0.161	0.114	0.511
*FAIM2/BCDIN3*	12	rs7138803	A	A	**1.88 (1.05–** **3.37)**	**0.034**	**0.039**	**1.93 (1.03–** **3.61)**	**0.040**	**0.024**	1.76 (0.84– 3.65)	0.132	0.266	0.850
*BDNF*	11	rs6265	G	G	**1.33 (1.01–** **1.74)**	**0.044**	**0.043**	1.21 (0.89– 1.63)	0.222	0.231	**1.59 (1.09–** **2.32)**	**0.017**	**0.014**	0.269
*SH2B1*	16	rs7498665	G	G	1.12 (0.94– 1.33)	0.195	0.175	**1.21 (1.00–** **1.46)**	**0.047**	**0.050**	0.97 (0.78– 1.22)	0.799	0.955	0.230
*GNPDA2*	4	rs10938397	G	G	1.13 (0.95– 1.36)	0.117	0.183	1.18 (0.97– 1.44)	0.097	0.070	1.05 (0.82– 1.33)	0.709	0.946	0.443
*MC4R*	18	rs17782313	C	C	1.24 (0.89– 1.72)	0.198	0.206	0.98 (0.68– 1.42)	0.923	0.880	**1.85 (1.23–** **2.80)**	**0.003**	**0.012**	**0.050**
*KCTD15*	19	rs29941	C	C	1.13 (0.92– 1.38)	0.237	0.163	1.14 (0.92– 1.42)	0.234	0.052	1.13 (0.88– 1.46)	0.342	0.828	0.958
*SEC16B/RASAL2*	1	rs10913469	C	C	1.10 (0.88– 1.36)	0.410	0.413	1.24 (0.98– 1.56)	0.072	0.111	0.79 (0.57– 1.09)	0.151	0.276	0.070
*NEGR1*	1	rs2815752	T	T	1.08 (0.88–1.31)	0.468	0.451	1.08 (0.86– 1.34)	0.506	0.621	1.10 (0.84– 1.43)	0.495	0.342	0.923
*NPC1*	18	rs1805081	A	G	1.07 (0.85– 1.35)	0.585	0.567	1.15 (0.90– 1.48)	0.269	0.228	0.87 (0.64– 1.18)	0.396	0.338	0.130
*SFRF10/ETV5*	3	rs7647305	C	C	1.08 (0.81– 1.44)	0.601	0.607	1.19 (0.87– 1.64)	0.283	0.304	0.92 (0.64– 1.32)	0.646	0.729	0.293
*MAF*	16	rs1424233	A	A	1.04 (0.83– 1.32)	0.714	0.700	1.07 (0.84– 1.37)	0.567	0.516	0.92 (0.67– 1.26)	0.603	0.559	0.446
*PRL*	6	rs4712652	A	A	1.02 (0.82– 1.26)	0.891	0.958	1.10 (0.87– 1.39)	0.424	0.704	0.86 (0.64– 1.15)	0.310	0.554	0.195
*MTCH2*	11	rs10838738	G	G	1.03 (0.83– 1.28)	0.764	0.958	1.06 (0.84– 1.33)	0.651	0.651	1.00 (0.76– 1.32)	0.991	0.971	0.782
*PTER*	10	rs10508503	C	T	1.03 (0.55– 1.93)	0.919	0.918	1.17 (0.61– 2.26)	0.630	0.605	0.77 (0.30– 1.94)	0.572	0.613	0.960

Chr, chromosome; CI, confidence interval; OR, odds ratio; *P*-Het, *P*-heterogeneity. SNPs were ranked by *P*-values. Statistically significant associations are bold-faced. ^a^ *P*-values were adjusted for age and sex, and ^b^ *P*-values were further adjusted for admixture. All *P*-values were tested under an additive model (*P*_add_), except those reported for *ADIPOQ*, *BCDIN3/FAIM2* and *BDNF* genes which were analyzed under a recessive model (*P*_rec_).

On the other hand, 5 of the 17 SNPs previously identified as obesity risk alleles by GWAS showed overall significant associations with obesity in the Mexican population (Table 1): *FTO* rs9939609 (OR 1.42, *P_add_*  = 0.001), *TMEM18* rs6548238 (OR 1.57, *P_add_*  = 0.003), *INSIG2* rs7566605 (OR 1.33 *P_add_*  = 0.006), *FAIM2*/*BCDIN3* rs7138803 (OR 1.88, *P_rec_*  = 0.034), and *BDNF* rs6265 (OR 1.33 *P_rec_*  = 0.044). On stratifying by obesity class, *SH2B1* rs7498665 was associated with class I/II obesity (OR 1.21, *P_add_*  = 0.047), and *MC4R* rs17782313 was associated with class III obesity (OR 1.85 *P_add_*  = 0.003). Interestingly, although *FTO* showed an overall association with obesity, the strongest and most significant association was observed for class III obesity (*P*_add_  = 4**×**10**^−^** ^6^). Heterogeneity in effect sizes between class I/II and class III obesity was statistically significant only for *FTO* rs9939609 (*P_het_*  = 0.031) and borderline significant for *MC4R* rs17782313 (*P_het_*  = 0.050). All risk alleles significantly associated with obesity were consistent with those previously reported, except for *INSIG2* rs7566605 as the risk allele is “C” in Europeans and “G” in the Mexican population. All associations remained significant after adjusting for admixture, except for that with the *UCP3* locus (Table 1).

When we examined the joint effects of the nine SNPs nominally associated with obesity, there was a significant increase in obesity risk with increasing mean number or risk alleles (±SD) adjusted for age, sex and admixture. Mean number of risk alleles was lower in normal weight (5.04±1.49) than in class I/II obese (5.36±1.53) and class III obese individuals (5.49±1.59) (OR 1.16 95% CI 1.04–1.29, *P* = 0.007 and OR 1.22 95% CI 1.06–1.40, *P* = 0.006, respectively).

### Cohort Studies: Associations with obesity-related traits in adults and children

We genotyped 12 SNPs in the 3 independent cohorts samples, including 9 SNPs showing significant association with obesity in the case-control study, plus 3 SNPs with P<0.20 in order to include polymorphisms which may have not reached nominal significance because of weaker effect and/or reduced sample size. Table 2 summarizes the results of associations with BMI and WC in each cohort and in the combined sample. Four loci (*FAIM2*/*BCDIN3*, *TMEM18*, *INSIG2* and *KCT15*) were significantly associated with BMI in adults, while only 2 loci (*FAIM2*/*BCDIN3* and *GNPDA2*) were significantly associated with BMI in children. In the combined analysis of Mexican-mestizo children and adults, 5 SNPs located at or near *FAIM2*/*BCDIN3*, *TMEM18*, *INSIG2*, *GNPDA2* and *SEC16B*/*RASAL2* were significantly associated with BMI and/or WC (P<0.05). Three of these genes (*FAIM2*/*BCDIN3*, *TMEM18*, and *INSIG2*) also showed the strongest associations with obesity in the case-control study. *FAIM2*/*BCDIN3* rs7138803 showed the strongest and most significant effect on BMI in both children and adults. The presence of two risk A allele copies represent an increase of 0.505 SD unit of BMI equivalent to 2.6 kg/m^2^ in adults (*P_rec_*  = 0.001), and a increase of 0.334 SD unit of BMI equivalent to 1.48 kg/m^2^ in children (*P_rec_*  = 0.008). Of note, *FTO* and *MC4R* variants were not significantly associated with BMI/WC variation in children, adults or in the combined analysis, which is consistent with their association mainly with class III obesity.

**Table 2:** Associations of 12 loci with BMI and WC in Mexican Adults and Children.

						Adults (n = 945)			Children (n = 1218)		All subjects (n = 2163)		
Trait	Nearest gene	SNP	Chr	Risk allele	RAF	Effect size (SE)	*P*	RAF	Effect size (SE)	*P*	Effect size (SE)	*P*	*P*-Het
**BMI**	*ADIPOQ*	rs2241766	3	G	17.6	0.14 (0.18)	0.420	18.2	0.05 (0.16)	0.757	0.09 (0.12)	0.442	0.695
	*UCP3*	rs1800849	11	T	12.8	−0.01 (0.07)	0.855	11.2	0.03 (0.061)	0.597	0.01 (0.05)	0.789	0.625
	*FTO*	rs9939609	16	A	19.4	0.05 (0.06)	0.392	18.1	0.08 (0.05)	0.125	0.07 (0.04)	0.085	0.757
	*TMEM18*	rs6548238	2	C	92.5	**0.25 (0.09)**	**0.005**	91.1	0.09 (0.07)	0.149	**0.15 (0.05)**	**0.004**	0.166
	*INSIG2*	rs7566605	2	G	76.6	**0.15 (0.06)**	**0.007**	73.4	0.03 (0.05)	0.475	**0.08 (0.04)**	**0.024**	0.095
	*FAIM2/ BCDIN3*	rs7138803	12	A	20.8	**0.51 (0.15)**	**0.001**	21.2	**0.33 (0.13**)	**0.008**	**0.40 (0.09)**	**3.5× 10^−5^**	0.388
	*BDNF*	rs6265	11	G	86.4	0.03 (0.08)	0.684	84.6	0.02 (0.06)	0.728	0.03 (0.05)	0.598	0.917
	*GNPDA2*	rs10938397	4	G	35.6	0.08 (0.05)	0.106	35.2	0.09 (0.04)	**0.021**	**0.09 (0.03)**	**0.005**	0.810
	*SH2B1*	rs7498665	16	G	49.9	0.06 (0.05)	0.181	50.3	−0.03 (0.04)	0.447	0.01 (0.03)	0.787	0.127
	*MC4R*	rs17782313	18	C	7.3	−0.04 (0.09)	0.690	8.2	−0.04 (0.07)	0.607	−0.04 (0.06)	0.515	0.993
	*KCTD15*	rs29941	19	C	62.6	0.11 (0.05)	0.020	55.2	0.02 (0.04)	0.658	0.054 (0.03)	0.072	0.121
	*SEC16B /RASAL2*	rs10913469	1	C	20.4	0.07 (0.06)	0.210	20.5	0.06 (0.05)	0.243	0.06 (0.04)	0.090	0.861
**WC**	*ADIPOQ*	rs2241766	3	G	17.6	0.01 (0.17)	0.944	18.2	0.15 (0.16)	0.339	0.09 (0.12)	0.451	0.555
	*UCP3*	rs1800849	11	T	12.8	−0.05 (0.07)	0.431	11.2	0.05 (0.06)	0.402	0.01 (0.04)	0.931	0.253
	*FTO*	rs9939609	16	A	19.4	0.05 (0.06)	0.394	18.1	0.04 (0.05)	0.354	0.05 (0.04)	0.209	0.936
	*TMEM18*	rs6548238	2	C	92.5	0.31 (0.09)	**3.4× 10^−4^**	91.1	0.08 (0.07)	0.200	**0.17 (0.05)**	**0.001**	**0.038**
	*INSIG2*	rs7566605	2	G	76.6	0.15 (0.05)	**0.007**	73.4	0.04 (0.04)	0.321	**0.08 (0.03)**	**0.014**	0.143
	*FAIM2 /BCDIN3*	rs7138803	12	A	20.8	0.62 (0.15)	**3.3× 10^−5^**	21.2	**0.28 (0.12)**	**0.024**	**0.42 (0.09)**	**1.1× 10^−5^**	0.082
	*BDNF*	rs6265	11	G	86.4	−0.03 (0.07)	0.692	84.6	0.04(0.06 )	0.450	0.02 (0.05)	0.741	0.437
	*GNPDA2*	rs10938397	4	G	35.6	0.11 (0.05)	0.021	35.2	0.04 (0.04)	0.304	**0.07 (0.03)**	**0.023**	0.274
	*SH2B1*	rs7498665	16	G	49.9	0.07 (0.04)	0.106	50.3	−0.05 (0.04)	0.229	0.01 (0.03)	0.903	0.042
	*MC4R*	rs17782313	18	C	7.3	0.03 (0.09)	0.770	8.2	−0.03 (0.07)	0.698	−0.01 (0.05)	0.908	0.639
	*KCTD15*	rs29941	19	C	62.6	0.08 (0.05)	0.074	55.2	0.03 (0.04)	0.449	0.05 (0.03)	0.082	0.365
	*SEC16B /RASAL2*	rs10913469	1	C	20.4	0.09 (0.05)	0.099	20.5	0.06 (0.05)	0.193	0.07 (0.03)	0.038	0.698

Abbreviations: Chr, chromosome; RAF, risk allele frequency; SE, standard error; *P*-Het, *P*-heterogeneity; BMI, body mass index; WC, waist circumference. Effect values are presented as effect size per allele copy, except for *ADIPOQ, BCDIN3*/*FAIM2* and *BDNF* analyzed under a recessive model, where effect size is reported for two allele copies. *P*-values were adjusted for age and sex. Statistically significant associations are bold-faced.

While there was no heterogeneity in effect size on BMI between adult and children populations, the effect size of *TMEM18* rs6548238 on waist circumference was significantly heterogeneous (*P_het_*  = 0.038) as it was approximately 3 fold higher in adults than in children (Table 2). Interestingly, although *SH2B1* rs7498665 was not significantly associated with waist circumference in children or adults, heterogeneity in effect size was statistically significant (*P_het_*  = 0.042), as adults bearing the “G” allele showed increased waist circumference, while children bearing this allele showed decreased waist circumference.

Table S2 shows associations of the 12 gene variants analyzed with biochemical measurements in children and adults. No significant associations of these SNPs with biochemical parameters were observed in the combined analysis. However, in adults the *MC4R* rs17782313 obesity-risk allele was associated with increased glucose levels (*P_add_*  = 0.034), *TMEM18* rs6548238 and *FTO* rs9939609 were associated with lower fasting insulin levels (*P_add_*  = 0.013 and 0.050, respectively), while *FTO* was associated with lower triglyceride levels (*P_add_*  = 0.048) and *BDNF* was associated with higher triglyceride levels (*P_rec_*  = 0.047) In children, only *INSIG2* and *BDNF* were significantly associated with higher and lower triglyceride levels (*P_add_*  = 0.004 and *P_rec_*  = 0.006, respectively).

### Association with obesity-related traits in Mexican Indigenous Populations

The 12 selected SNPs were genotyped in a total of 543 indigenous Mexicans. Overall, most obesity-risk alleles were less frequent in this group than in Mexican Mestizo and European populations. Comparisons of risk allele frequencies among European, Mestizo and Indigenous Mexican populations are shown in Table S3. Interestingly, only *FTO* rs9939609 was significantly associated with increased BMI, and each copy of the risk A allele increases 0.250 SD unit of BMI equivalent to 1.22 kg/m^2^ (*P_add_*  = 0.045; Table 3).

**Table 3:** Association of 12 loci with BMI in Indigenous Mexican population.

Trait	Nearest gene	SNP	Chr	Risk allele	RAF	Effect size (SE)	*P*
**BMI**	*ADIPOQ*	rs2241766	3	G	20.34	−0.16 (0.22)	0.454
	*UCP3*	rs1800849	11	C	10.90	−0.02 (0.09)	0.808
	*FTO*	rs9939609	16	A	5.50	**0.25 (0.13)**	**0.045**
	*TMEM18*	rs6548238	2	C	94.24	0.07 (0.13)	0.562
	*INSIG2*	rs7566605	2	G	77.04	−0.02 (0.07)	0.804
	*FAIM2/* *BCDIN3*	rs7138803	12	A	16.42	0.05 (0.27)	0.844
	*BDNF*	rs6265	11	G	89.50	0.06 (0.10)	0.525
	*GNPDA2*	rs10938397	4	G	29.58	−0.01 (0.06)	0.864
	*SH2B1*	rs7498665	16	G	44.01	0.06 (0.06)	0.310
	*MC4R*	rs17782313	18	C	1.52	0.36 (0.20)	0.077
	*KCTD15*	rs29941	19	C	52.53	−0.04 (0.06)	0.518
	*SEC16B* */RASAL2*	rs10913469	1	C	27.22	−0.07 (0.07)	0.308

Abbreviations: Chr, chromosome; RAF, risk allele frequency; SE, standard error; *P*-Het, *P*-heterogeneity; BMI, body mass index. Effect values are presented as effect size per allele copy, except for *ADIPOQ*, *BCDIN3*/*FAIM2* and *BDNF* analyzed under a recessive model, where effect size is reported for two allele copies. *P*-values were adjusted for age and sex. Statistically significant associations are bold-faced.

## Discussion

In the present study, we analyzed 26 SNPs previously associated with obesity in a case-control study in adult Mexican Mestizo subjects. In this population, only 2 of the 8 (25%) biological candidate genes (*ADIPOQ* and *UCP3*) were associated with obesity, although only *ADIPOQ* remained significant after adjusting for admixture. Given the substantial number of obesity association replications in various ethnicities [5], it is noteworthy that the proportion of these biological candidate genes associated with obesity in the present study was low. However, this is consistent with several other studies failing to show associations with such genes, and the fact that these genes are not significantly associated with obesity in most GWAS [16]–[18]. In addition, 7 of the 17 (41.2%) GWAs-selected genes (*FTO*, *TMEM18*, *INSIG2*, *FAIM2*/*BCDIN3*, *BDNF*, *SH2B1* and *MC4R*) were associated with obesity in this population. This proportion is similar to that reported in other studies seeking to replicate associations of these variants with obesity [29]–[31]. Thus, it is clear that some of the previously obesity-associated variants identified in Europeans also confer susceptibility to obesity in Mexican population. Interestingly, the significant ORs observed here were higher, although *P* values were lower than those reported in the original studies [12]–[18], which may be explained by the smaller sample size analyzed in this study.

*FTO*, *TMEM18* and *INSIG2* variants showed the most significant associations with obesity in this population. *FTO* and *TMEM18* are probably the most replicated genes showing the strongest and most significant effects in various different populations [30], [32]–[34]. One of the most notable results is the association of obesity with *INSIG2*, as previous studies have shown conflicting results and few studies have replicated this association [35]–[38]. Moreover, in contrast to the original report of Herbert et al. [12], we found that the G and not the C allele confers risk for obesity. Linkage disequilibrium with other variants may explain this inverse association.

Interestingly, *FTO*, *MC4R* and *BDNF* loci were most significantly associated with class III obesity. The association of *FTO* rs9939609 with class III obesity has been previously observed in the Mexican and several other populations [8], [14], [39], [40]. On the other hand, *MC4R* and *BDNF* mutations are known to cause monogenic obesity [41], [42], and common variation in these genes has also been previously associated with severe obesity [15]–[18]. The more significant association with class III obesity may be explained by the presence of rare functional variants in high LD with the variants studied here.

Seventeen of the 25 SNPs analyzed failed to show associations with obesity in the Mexican population. However, the trend of association for most GWAS-selected SNPs was in the same direction as the initially reported findings. Considering that ORs reported in Europeans were around 1.1 for these 21 SNPs [5], [16]–[18], and that most risk alleles were less frequent in Mexican Mestizos than in Europeans, the lack of association observed here may be due to insufficient statistical power (range 6.3%–72.7% for 13 of these SNPs). Further studies in larger samples are necessary to confirm whether these 13 or other SNPs contribute to the risk of obesity in Mexicans.

Some of the obesity-associated genes in this study have known functions, *ADIPOQ* is known to decrease body weight by increasing lipid oxidation in muscles and other organs [43], and *UCP3* plays an important role in human energy homeostasis [44]. However, little is known about the mechanisms responsible for associations of the majority of GWAS loci with obesity. Because *FTO*, *TMEM18*, *MC4R*, *FAIM2*/*BCDIN3*, *KCTD15* and *GNPDA2* are specifically expressed in hypothalamic regions [16], [17], it has been suggested that the associations may result from neuronal effect on energy balance. However, many of these loci are located near multiple genes, and the causal variants need to be identified.

Although nominal significant associations of 12 SNPs with risk of obesity were found in the case-control study, not all were associated with BMI and/or WC in the population-based studies. Only the *FAIM2*/BCDIN3 variant was associated with both BMI and WC in children and adults, suggesting that the obesogenic effect of this variant is present since early ages. This is consistent with previous reports [45], [46]. In contrast, other SNPs showed differences according to age group. In adults, *TMEM18*, *INSIG2* and *KCTD15* were significantly associated with BMI, while in children only *GNPDA2* showed a significant association. The replication of the *GNPDA2* locus is consistent with a previous report in Mexican children [10].

It is noteworthy that, although *FTO* was most significantly associated with obesity in the case-control study, its association with BMI did not reach statistical significance in children, adults or in the combined analyses (*P*_add_  = 0.125, 0.392 and 0.085, respectively). However, the direction of effect in both groups was consistent with the previous observation on obesity risk in Mexicans [8]. The lack of association of *FTO* with BMI in children is in accordance with a longitudinal study on the life course effects of *FTO*, which is weaker in childhood and strengthens up to age 20 years [47]. Moreover, the lack of association of *FTO* with BMI in adults is consistent with its most significant association with class III obesity, and the fact that only 2% of the adults included in this cohort had this class of obesity. The latter may also explain the lack of association of *MC4R* and *BNDF* with BMI in adults.

Based on linear regression analyses, the 12 obesity-associated SNPs in the current study explained only 3.3% and 1.1% of the variation in BMI in adults and children. This suggests that many more common variants with small effects, and perhaps rare variants with larger effect, remain to be identified to account for even the lower end of estimated heritability of BMI in Mexican populations (range 36–62%) [48], [49]. Because genes are non-modifiable risk factors, the identification of obesity-related genes is only the first step in an attempt to understand this complex disease. Gene-environment or gene-diet interactions need to be explored to provide information that may help establish preventive measures.

Interestingly, we confirmed the effect of the *FTO* locus on obesity in adult indigenous Mexican populations, despite of lower frequency of risk allele as compared to Europeans. This finding is consistent with previous observations in Pima Indians [50]. For the remaining 11 obesity loci analyzed, no association with BMI was observed. Differences in allele frequencies and LD between populations may have masked possible associations. Moreover, due to the small effects reported for these variants, the sample size of our study may not have the power to detect all associations.

In conclusion, this is the first study analyzing several obesity-associated SNPs in Mexican adults, children and Indigenous populations. The study replicates the association of 8 SNPs with obesity risk in Mexican adults, and confirms the role of some of these SNPs in BMI of Mexican mestizo children and adults, and Mexican Indigenous populations. Our results suggest that obesity risk loci identified in Europeans also confer risk for obesity and increased BMI in Mexicans, and that diverse ethnic groups share some degree of genetic predisposition to obesity.

## Supporting Information

Table S1 **Anthropometric characteristics of case and control subjects.**
(DOC)

Table S2 **Associations of 12 loci with Biochemical Characteristics in Mexican Adults and Children.**
(DOC)

Table S3 **Comparison of Risk Allele Frequencies of 12 SNPs in Mexican Indigenous, Mexican Mestizo and Caucasian populations.**
(DOC)
