# Supplementary material for: Contribution of Common Genetic Variants to Obesity and Obesity-Related Traits in Mexican Children and Adults
Source: PLoS One. 2013 Aug 8;8(8):e70640. doi: 10.1371/journal.pone.0070640 (PMC3738539; doi:10.1371/journal.pone.0070640)
Supplement: Table S1 — Anthropometric characteristics of case and control subjects. (DOC) [file pone.0070640.s001.doc]

**Table S1**. Anthropometric characteristics of case and control subjects.

|  | Normal weight | Class I/II obese | Class III obese | P-value |
| --- | --- | --- | --- | --- |
| *n* | 473 | 441 | 242 |  |
| Male (%) | 35.9 | 31.9 | 26.0 | <0.001 |
| Age (years) | 38.4±13.9 | 42.5±13.1 | 39.4±11.0 | 0.271 |
| BMI (kg/m2) | 22.8±1.9 | 33.4±2.9 | 46.7±5.9 | <0.001 |
| WC (cm) | 79.4±8.5 | 103.9±10.6 | 129.7±15.8 | <0.001 |

BMI, body mass index; WC, waist circumference. Categorical data are expressed as percentage. Differences between groups were assessed by the Chi-square test; continuous variables are shown as mean ± SD and were assessed by ANOVA test.
