# Supplementary material for: Contribution of Common Genetic Variants to Obesity and Obesity-Related Traits in Mexican Children and Adults
Source: PLoS One. 2013 Aug 8;8(8):e70640. doi: 10.1371/journal.pone.0070640 (PMC3738539; doi:10.1371/journal.pone.0070640)
Supplement: Table S2 — Associations of 12 loci with Biochemical Characteristics in Mexican Adults and Children. (DOC) [file pone.0070640.s002.doc]

**Table S2**. Associations of 12 loci with Biochemical Characteristics in Mexican Adults and Children

|  |  |  |  |  |  | Adults (n=945) | |  |  | Children (n=1218) | |  | All subjects (n=2163) | |
| --- | --- | --- | --- | --- | --- | --- | --- | --- | --- | --- | --- | --- | --- | --- |
| Trait | Nearest | SNP | Chr | Risk | RAF | Effect size | P |  | RAF | Effect size | *P* |  | Effect size | *P* |
|  | gene |  |  | allelic |  | (SE) |  |  |  | (SE) |  |  | (SE) |  |
| **Fasting Glucose** | *ADIPOQ* | rs2241766 | 3 | G | 17.6 | -0.05 (0.18) | 0.779 |  | 18.2 | -0.02 (0.17) | 0.929 |  | -0.03 (0.12) | 0.797 |
|  | *UCP3* | rs1800849 | 11 | C | 12.8 | -0.03 (0.07) | 0.692 |  | 11.2 | 0.05 (0.06) | 0.415 |  | 0.01 (0.05) | 0.745 |
|  | *FTO* | rs9939609 | 16 | A | 19.4 | 0.04 (0.06) | 0.516 |  | 18.1 | 0.04 (0.05) | 0.424 |  | 0.04 (0.04) | 0.301 |
|  | *TMEM18* | rs6548238 | 2 | C | 92.5 | 0.01 (0.09) | 0.951 |  | 91.1 | 0.04 (0.07) | 0.582 |  | 0.03 (0.05) | 0.637 |
|  | *INSIG2* | rs7566605 | 2 | G | 76.6 | -0.09 (0.06) | 0.116 |  | 73.4 | -0.05 (0.05) | 0.248 |  | -0.07 (0.04) | 0.059 |
|  | *FAIM2/BCDIN3* | rs7138803 | 12 | A | 20.8 | -0.03 (0.16) | 0.873 |  | 21.2 | 0.03 (0.13) | 0.794 |  | 0.01 (0.10) | 0.925 |
|  | *BDNF* | rs6265 | 11 | G | 86.4 | 0.02 (0.08) | 0.83 |  | 84.6 | -0.03 (0.06) | 0.598 |  | -0.01 (0.05) | 0.788 |
|  | *GNPDA2* | rs10938397 | 4 | G | 35.6 | -0.03 (0.05) | 0.576 |  | 35.2 | -0.03 (0.04) | 0.496 |  | -0.03 (0.03) | 0.376 |
|  | *SH2B1* | rs7498665 | 16 | G | 49.9 | 0.02 (0.05) | 0.671 |  | 50.3 | -0.01 (0.04) | 0.930 |  | 0.01 (0.03) | 0.845 |
|  | *MC4R* | rs17782313 | 18 | C | 7.3 | 0.19 (0.09) | 0.034 |  | 8.2 | 0.02 (0.07) | 0.811 |  | 0.09 (0.06) | 0.119 |
|  | *KCTD15* | rs29941 | 19 | C | 62.6 | 0.04 (0.05) | 0.462 |  | 55.2 | -0.01 (0.04) | 0.880 |  | 0.01 (0.03) | 0.707 |
|  | *SEC16B/RASAL2* | rs10913469 | 1 | C | 20.4 | 0.08 (0.06) | 0.17 |  | 20.5 | -0.05 (0.05) | 0.303 |  | 0.01 (0.04) | 0.866 |
| **Fasting Insulin** | *ADIPOQ* | rs2241766 | 3 | G | 17.6 | -0.11 (0.18) | 0.538 |  | 18.2 | -0.07 (0.25) | 0.794 |  | -0.10 (0.15) | 0.512 |
|  | *UCP3* | rs1800849 | 11 | C | 12.8 | -0.04 (0.07) | 0.537 |  | 11.2 | -0.04 (0.09) | 0.629 |  | -0.04 (0.05) | 0.436 |
|  | *FTO* | rs9939609 | 16 | A | 19.4 | **-0.14 (0.06)** | **0.013** |  | 18.1 | 0.01 (0.07) | 0.912 |  | -0.08 (0.04) | 0.062 |
|  | *TMEM18* | rs6548238 | 2 | C | 92.5 | **-0.17 (0.09)** | **0.05** |  | 91.1 | -0.02 (0.10) | 0.846 |  | -0.10 (0.06) | 0.113 |
|  | *INSIG2* | rs7566605 | 2 | G | 76.6 | -0.05 (0.05) | 0.309 |  | 73.4 | -0.03 (0.06) | 0.578 |  | -0.04 (0.04) | 0.253 |
|  | *FAIM2/BCDIN3* | rs7138803 | 12 | A | 20.8 | 0.13 (0.15) | 0.379 |  | 21.2 | 0.04 (0.17) | 0.830 |  | 0.09 (0.11) | 0.422 |
|  | *BDNF* | rs6265 | 11 | G | 86.4 | 0.13 (0.07) | 0.071 |  | 84.6 | -0.03 (0.08) | 0.720 |  | 0.06 (0.05) | 0.255 |
|  | *GNPDA2* | rs10938397 | 4 | G | 35.6 | -0.01 (0.05) | 0.91 |  | 35.2 | 0.02 (0.05) | 0.645 |  | 0.01 (0.03) | 0.835 |
|  | *SH2B1* | rs7498665 | 16 | G | 49.9 | -0.01 (0.05) | 0.85 |  | 50.3 | 0.02 (0.05) | 0.716 |  | 0.01 (0.03) | 0.921 |
|  | *MC4R* | rs17782313 | 18 | C | 7.3 | 0.03 (0.09) | 0.766 |  | 8.2 | -0.01 (0.10) | 0.965 |  | 0.01 (0.07) | 0.843 |
|  | *KCTD15* | rs29941 | 19 | C | 62.6 | -0.01 (0.05) | 0.902 |  | 55.2 | 0.03 (0.05) | 0.527 |  | 0.01 (0.03) | 0.741 |
|  | *SEC16B-RASAL2* | rs10913469 | 1 | C | 20.4 | -0.01 (0.06) | 0.826 |  | 20.5 | 0.02 (0.07) | 0.755 |  | 0.01 (0.04) | 0.971 |
| **HOMA-IR** | *ADIPOQ* | rs2241766 | 3 | G | 17.6 | -0.11 (0.19) | 0.583 |  | 18.2 | -0.08 (0.26) | 0.749 |  | -0.10 (0.16) | 0.53 |
|  | *UCP3* | rs1800849 | 11 | C | 12.8 | -0.06 (0.07) | 0.414 |  | 11.2 | -0.01 (0.09) | 0.866 |  | -0.04 (0.05) | 0.458 |
|  | *FTO* | rs9939609 | 16 | A | 19.4 | -0.10 (0.06) | 0.117 |  | 18.1 | 0.04 (0.07) | 0.574 |  | -0.04 (0.05) | 0.416 |
|  | *TMEM18* | rs6548238 | 2 | C | 92.5 | -0.12 (0.09) | 0.213 |  | 91.1 | -0.01 (0.10) | 0.894 |  | -0.07 (0.07) | 0.322 |
|  | *INSIG2* | rs7566605 | 2 | G | 76.6 | -0.09 (0.05) | 0.09 |  | 73.4 | -0.03 (0.06) | 0.581 |  | -0.07 (0.04) | 0.103 |
|  | *FAIM2/BCDIN3* | rs7138803 | 12 | A | 20.8 | 0.07 (0.16) | 0.655 |  | 21.2 | 0.06 (0.17) | 0.739 |  | 0.06 (0.12) | 0.58 |
|  | *BDNF* | rs6265 | 11 | G | 86.4 | 0.11 (0.08) | 0.134 |  | 84.6 | -0.04 (0.08) | 0.658 |  | 0.05 (0.06) | 0.414 |
|  | *GNPDA2* | rs10938397 | 4 | G | 35.6 | 0.01 (0.05) | 0.831 |  | 35.2 | 0.03 (0.05) | 0.574 |  | 0.02 (0.04) | 0.598 |
|  | *SH2B1* | rs7498665 | 16 | G | 49.9 | -0.01 (0.05) | 0.905 |  | 50.3 | 0.02 (0.05) | 0.660 |  | 0.01 (0.03) | 0.838 |
|  | *MC4R* | rs17782313 | 18 | C | 7.3 | 0.04 (0.09) | 0.644 |  | 8.2 | -0.01 (0.10) | 0.946 |  | 0.02 (0.07) | 0.763 |
|  | *KCTD15* | rs29941 | 19 | C | 62.6 | -0.01 (0.05) | 0.972 |  | 55.2 | 0.03 (0.05) | 0.501 |  | 0.01 (0.04) | 0.672 |
|  | *SEC16B/RASAL2* | rs10913469 | 1 | C | 20.4 | 0.01 (0.06) | 0.822 |  | 20.5 | 0.01 (0.07) | 0.857 |  | 0.01 (0.04) | 0.778 |
| **Total Cholesterol** | *ADIPOQ* | rs2241766 | 3 | G | 17.6 | -0.08 (0.17) | 0.628 |  | 18.2 | 0.05 (0.16) | 0.751 |  | -0.01 (0.11) | 0.92 |
|  | *UCP3* | rs1800849 | 11 | C | 12.8 | 0.07 (0.06) | 0.281 |  | 11.2 | 0.01 (0.06) | 0.997 |  | 0.03 (0.04) | 0.446 |
|  | *FTO* | rs9939609 | 16 | A | 19.4 | -0.06 (0.06) | 0.259 |  | 18.1 | 0.06 (0.05) | 0.752 |  | -0.02 (0.04) | 0.621 |
|  | *TMEM18* | rs6548238 | 2 | C | 92.5 | -0.03 (0.08) | 0.703 |  | 91.1 | 0.04 (0.06) | 0.573 |  | 0.01 (0.05) | 0.833 |
|  | *INSIG2* | rs7566605 | 2 | G | 76.6 | 0.06 (0.05) | 0.245 |  | 73.4 | 0.04 (0.04) | 0.345 |  | 0.05 (0.03) | 0.141 |
|  | *FAIM2/BCDIN3* | rs7138803 | 12 | A | 20.8 | -0.07 (0.14) | 0.648 |  | 21.2 | 0.21 (0.12) | 0.087 |  | 0.09 (0.09) | 0.32 |
|  | *BDNF* | rs6265 | 11 | G | 86.4 | -0.04 (0.07) | 0.542 |  | 84.6 | -0.07 (0.06) | 0.262 |  | -0.06 (0.04) | 0.211 |
|  | *GNPDA2* | rs10938397 | 4 | G | 35.6 | 0.04 (0.04) | 0.433 |  | 35.2 | -0.08 (0.04) | 0.041 |  | -0.03 (0.03) | 0.336 |
|  | *SH2B1* | rs7498665 | 16 | G | 49.9 | -0.01 (0.04) | 0.799 |  | 50.3 | -0.05 (0.04) | 0.192 |  | -0.03 (0.03) | 0.256 |
|  | *MC4R* | rs17782313 | 18 | C | 7.3 | 0.04 (0.08) | 0.606 |  | 8.2 | 0.07 (0.07) | 0.321 |  | 0.06 (0.05) | 0.272 |
|  | *KCTD15* | rs29941 | 19 | C | 62.6 | -0.05 (0.05) | 0.306 |  | 55.2 | 0.02 (0.04) | 0.549 |  | -0.01 (0.03) | 0.844 |
|  | *SEC16B/RASAL2* | rs10913469 | 1 | C | 20.4 | 0.04 (0.05) | 0.468 |  | 20.5 | -0.07 (0.05) | 0.143 |  | -0.02 (0.03) | 0.554 |
| **Triglycerides** | *ADIPOQ* | rs2241766 | 3 | G | 17.6 | -0.14 (0.18) | 0.439 |  | 18.2 | 0.16 (0.16) | 0.322 |  | 0.02 (0.12) | 0.848 |
|  | *UCP3* | rs1800849 | 11 | C | 12.8 | -0.01 (0.07) | 0.906 |  | 11.2 | -0.06 (0.06) | 0.343 |  | -0.03 (0.05) | 0.436 |
|  | *FTO* | rs9939609 | 16 | A | 19.4 | **-0.12 (0.06)** | **0.048** |  | 18.1 | 0.02 (0.05) | 0.608 |  | -0.03 (0.04) | 0.382 |
|  | *TMEM18* | rs6548238 | 2 | C | 92.5 | 0.06 (0.09) | 0.514 |  | 91.1 | 0.09 (0.07) | 0.180 |  | 0.08 (0.05) | 0.145 |
|  | *INSIG2* | rs7566605 | 2 | G | 76.6 | -0.02 (0.05) | 0.653 |  | 73.4 | **0.13 (0.04)** | **0.004** |  | 0.07 (0.03) | 0.051 |
|  | *FAIM2/BCDIN3* | rs7138803 | 12 | A | 20.8 | -0.05 (0.15) | 0.747 |  | 21.2 | 0.03 (0.13) | 0.785 |  | 0.01 (0.10) | 0.999 |
|  | *BDNF* | rs6265 | 11 | G | 86.4 | **0.15 (0.07)** | **0.047** |  | 84.6 | **-0.17 (0.06)** | **0.006** |  | -0.04 (0.05) | 0.394 |
|  | *GNPDA2* | rs10938397 | 4 | G | 35.6 | 0.02 (0.05) | 0.584 |  | 35.2 | -0.06 (0.04) | 0.161 |  | -0.02 (0.03) | 0.499 |
|  | *SH2B1* | rs7498665 | 16 | G | 49.9 | 0.02 (0.04) | 0.603 |  | 50.3 | -0.06 (0.04) | 0.101 |  | -0.03 (0.03) | 0.376 |
|  | *MC4R* | rs17782313 | 18 | C | 7.3 | -0.04 (0.09) | 0.669 |  | 8.2 | 0.05 (0.07) | 0.480 |  | 0.01 (0.05) | 0.784 |
|  | *KCTD15* | rs29941 | 19 | C | 62.6 | -0.03 (0.05) | 0.479 |  | 55.2 | -0.05 (0.04) | 0.174 |  | -0.04 (0.03) | 0.133 |
|  | *SEC16B/RASAL2* | rs10913469 | 1 | C | 20.4 | 0.08 (0.06) | 0.156 |  | 20.5 | 0.01 (0.05) | 0.839 |  | 0.04 (0.04) | 0.276 |
| **HDL-Cholesterol** | *ADIPOQ* | rs2241766 | 3 | G | 17.6 | 0.16 (0.16) | 0.311 |  | 18.2 | 0.01 (0.16) | 0.926 |  | 0.09 (0.11) | 0.437 |
|  | *UCP3* | rs1800849 | 11 | C | 12.8 | 0.04 (0.06) | 0.531 |  | 11.2 | 0.12 (0.06) | 0.052 |  | 0.08 (0.04) | 0.069 |
|  | *FTO* | rs9939609 | 16 | A | 19.4 | 0.11 (0.05) | 0.049 |  | 18.1 | 0.02 (0.05) | 0.695 |  | 0.06 (0.04) | 0.11 |
|  | *TMEM18* | rs6548238 | 2 | C | 92.5 | -0.06 (0.08) | 0.467 |  | 91.1 | -0.06 (0.07) | 0.321 |  | -0.06 (0.05) | 0.222 |
|  | *INSIG2* | rs7566605 | 2 | G | 76.6 | 0.01 (0.05) | 0.989 |  | 73.4 | -0.05 (0.04) | 0.295 |  | -0.03 (0.03) | 0.436 |
|  | *FAIM2/BCDIN3* | rs7138803 | 12 | A | 20.8 | 0.09 (0.14) | 0.504 |  | 21.2 | 0.12 (0.12) | 0.324 |  | 0.11 (0.09) | 0.239 |
|  | *BDNF* | rs6265 | 11 | G | 86.4 | -0.03 (0.07) | 0.061 |  | 84.6 | 0.02 (0.06) | 0.734 |  | -0.01 (0.04) | 0.99 |
|  | *GNPDA2* | rs10938397 | 4 | G | 35.6 | -0.06 (0.04) | 0.184 |  | 35.2 | -0.01 (0.04) | 0.750 |  | -0.03 (0.03) | 0.254 |
|  | *SH2B1* | rs7498665 | 16 | G | 49.9 | -0.04 (0.04) | 0.289 |  | 50.3 | 0.03 (0.04) | 0.500 |  | -0.01 (0.03) | 0.82 |
|  | *MC4R* | rs17782313 | 18 | C | 7.3 | -0.05 (0.08) | 0.563 |  | 8.2 | 0.03 (0.07) | 0.712 |  | -0.01 (0.05) | 0.915 |
|  | *KCTD15* | rs29941 | 19 | C | 62.6 | 0.01 (0.04) | 0.868 |  | 55.2 | 0.02 (0.04) | 0.610 |  | 0.01 (0.03) | 0.615 |
|  | *SEC16B/RASAL2* | rs10913469 | 1 | C | 20.4 | 0.01 (0.05) | 0.913 |  | 20.5 | -0.01 (0.05) | 0.895 |  | -0.01 (0.03) | 0.995 |

Abbreviations: Chr, chromosome; RAF, risk allele frequency; SE, standard error; *P*-Het, *P*-heterogeneity; HOMA-IR, homeostasis model assessment of insulin resistance; HDL, High-density lipoprotein. Effect values are presented as effect size per allele copy, except for *ADIPOQ, BCDIN3*/*FAIM2* and *BDNF* analyzed under a recessive model, where effect size is reported for two allele copies. *P*-values were adjusted for age and sex. Statistically significant associations are bold-faced.
