# Supplementary material for: Contribution of Common Genetic Variants to Obesity and Obesity-Related Traits in Mexican Children and Adults
Source: PLoS One. 2013 Aug 8;8(8):e70640. doi: 10.1371/journal.pone.0070640 (PMC3738539; doi:10.1371/journal.pone.0070640)
Supplement: Table S3 — Comparison of Risk Allele Frequencies of 12 SNPs in Mexican Indigenous, Mexican Mestizo and Caucasian populations. (DOC) [file pone.0070640.s003.doc]

**Table S3**. Comparison of Risk Allele Frequencies of 12 SNPs in Mexican Indigenous, Mexican Mestizo and Caucasian populations.

| Nearest gene | SNP | Chr | Risk allele | Risk allele frequency | | | | | |
| --- | --- | --- | --- | --- | --- | --- | --- | --- | --- |
|  |  |  |  | Mexican Indigenous | Mexican Mestizos | Caucasians | *^a^P* | *^b^P* | *^c^P* |
| *ADIPOQ* | rs2241766 | 3 | G | 0.203 | 0.179 | 0.136 | 0.070 | 0.013 | 0.108 |
| *UCP3* | rs1800849 | 11 | C | 0.109 | 0.119 | 0.239 | 0.128 | <0.001 | <0.001 |
| *FTO* | rs9939609 | 16 | A | 0.055 | 0.187 | 0.460 | <0.001 | <0.001 | <0.001 |
| *TMEM18* | rs6548238 | 2 | C | 0.942 | 0.917 | 0.850 | 0.072 | <0.001 | <0.001 |
| *INSIG2* | rs7566605 | 2 | G | 0.770 | 0.748 | 0.735 | 0.773 | 0.135 | 0.164 |
| *FAIM2/BCDIN3* | rs7138803 | 12 | A | 0.164 | 0.210 | 0.345 | <0.05 | <0.001 | <0.001 |
| *BDNF* | rs6265 | 11 | G | 0.895 | 0.854 | 0.805 | <0.05 | <0.001 | <0.05 |
| *GNPDA2* | rs10938397 | 4 | G | 0.296 | 0.354 | 0.446 | <0.05 | <0.001 | <0.05 |
| *SH2B1* | rs7498665 | 16 | G | 0.440 | 0.498 | 0.379 | <0.05 | < 0.05 | <0.001 |
| *MC4R* | rs17782313 | 18 | C | 0.015 | 0.078 | 0.266 | <0.001 | <0.001 | <0.001 |
| *KCTD15* | rs29941 | 19 | C | 0.525 | 0.584 | 0.690 | <0.001 | <0.001 | <0.05 |
| *SEC16B/RASAL2* | rs10913469 | 1 | C | 0.272 | 0.204 | 0.255 | <0.001 | 0.471 | <0.05 |

Abbreviations: Chr, chromosome. ^a^*P*-values for comparisons between Mexican Indigenous and Mexican Mestizos. ^b^*P*-values for comparisons between Mexican Indigenous and Caucasians. *^c^P*-values for comparisons between Mexican Mestizos and Caucasians.
